# Changes in brain metabolic connectivity underlie autistic-like social deficits in a rat model of autism spectrum disorder

**DOI:** 10.1038/s41598-017-13642-3

**Published:** 2017-10-16

**Authors:** Hojin Cho, Chul Hoon Kim, Elizabeth Quattrocki Knight, Hye Won Oh, Bumhee Park, Dong Goo Kim, Hae-Jeong Park

**Affiliations:** 1 Department of Pharmacology, Yonsei University College of Medicine, Seoul, Republic of Korea; 2 BK21 PLUS Project for Medical Science, Yonsei University College of Medicine, Seoul, Republic of Korea; 3 Department of Nuclear Medicine, Severance Hospital, Yonsei University College of Medicine, Seoul, Republic of Korea; 4 Brain Research Institute, Yonsei University College of Medicine, Seoul, Republic of Korea; 5 Severance Biomedical Science Institute, Yonsei University College of Medicine, Seoul, Republic of Korea; 6 Department of Psychiatry, Harvard Medical School, McLean Hospital, Belmont, MA USA; 7 Department of Statistics, Hankuk University of Foreign Studies, Yong-In, Republic of Korea; 8 Department of Psychiatry, Department of Cognitive Science, Yonsei University College of Medicine, Seoul, Republic of Korea

## Abstract

The neurobiological basis of social dysfunction and the high male prevalence in autism spectrum disorder (ASD) remain poorly understood. Although network alterations presumably underlie the development of autistic-like behaviors, a clear pattern of connectivity differences specific to ASD has not yet emerged. Because the heterogeneous nature of ASD hinders investigations in human subjects, we explored brain connectivity in an etiologically homogenous rat model of ASD induced by exposure to valproic acid (VPA) *in utero*. We performed partial correlation analysis of cross-sectional resting-state ^18^F-fluorodeoxyglucose positron emission tomography scans from VPA-exposed and control rats to estimate metabolic connectivity and conducted canonical correlation analysis of metabolic activity and behavior scores. VPA-treated rats exhibited impairments in social behaviors, and this difference was more pronounced in male than female rats. Similarly, current analyses revealed sex-specific changes in network connectivity and identified distinct alterations in the distributed metabolic activity patterns associated with autistic-like social deficits. Specifically, diminished activity in the salience network and enhanced activity in a cortico-cerebellar circuit correlated with the severity of social behavioral deficits. Such metabolic connectivity features may represent neurobiological substrates of autistic-like behavior, particularly in males, and may serve as a pathognomonic sign in the VPA rat model of ASD.

## Introduction

Autism spectrum disorder (ASD) is a lifelong neurodevelopmental disorder characterized by early-onset impairments in social communication and social interaction^1^. ASD comprises a highly heterogeneous set of disorders, with variable levels of functioning and comorbidity^2^. This diversity poses a considerable challenge for accurate diagnosis, especially in preverbal children^3^. ASD has a strong genetic heritability with complex inheritance^4,5^. In addition, many cases of ‘idiopathic’ ASD, have no known genetic causes and are likely affected by environmental factors^6^. The heterogeneous nature of the origins of ASD complicates the neurobiological exploration of this disorder^7,8^. Therefore, a well-controlled study in an etiologically homogeneous model of ASD would help mitigate this complexity.

A strong male predominance has been observed in ASD; boys are diagnosed five times more frequently than girls are. Sex-specific differences are thought to potentiate risk in males and/or attenuate risk in females induced by genetic and environmental factors^9^. Alternatively, or in addition, the different prevalence between boys and girls may arise from diagnostic biases^10,11^. However, little attention has been paid to how ASD in males differs from those in females, particularly how differences in neurobiology may underlie differences in behavior.

In order to help identify the neurobiological basis of ASD, particularly with regard to variability in social interactions and sex-specific manifestations, we explored the correlation between behaviors and specific alterations in neuronal connectivity in the rat model of ASD induced by prenatal exposure to valproic acid (VPA). VPA is one of the most commonly prescribed teratogenic drugs in women of child-bearing potential and prenatal exposure to VPA is known to be associated with an increased risk of ASD in humans^12–14^. Rats prenatally exposed to VPA displayed reduced social interactions, increased repetitive/stereotyped behaviors, and early signs of neurodevelopmental delay^15^. Previous animal studies also reported that VPA induced more extensive behavioral and molecular alterations in males than in females^16,17^. However, most previous studies have focused primarily on males, thus a clear understanding of sex differences has not emerged^18^.

In the present study, we regarded ASD as a disorder of abnormal neural connectivity^19–21^, and examined alterations in inter-regional connectivity across the entire brain in the VPA rat model of ASD. A growing body of evidence supports the notion that ASD is associated with altered brain connectivity^22,23^. Although decreases in white matter integrity and impaired long-range connectivity have been reported in humans with ASD^20,24^, prior studies frequently describe inconsistent findings and even contradictory results^25,26^. Thus, the connectivity changes associated with ASD and their relation to the behavioral phenotypes are still uncertain^26–28^. The VPA rat model mitigates the aforementioned influences of heterogeneous causality, and thereby provides an opportunity to reveal a more direct link between behavioral manifestations of ASD and changes in brain connectivity.

Among the plethora of methods for analyzing brain connectivity, we evaluated alterations in brain metabolic connectivity of VPA-treated and control rats using resting-state ^18^F-fluorodeoxyglucose (FDG) positron emission tomography (PET). Recent investigations regarding brain circuitry have begun to utilize resting-state functional connectivity mapping with functional magnetic resonance imaging (fMRI) in animals^29^. However, unlike studies involving humans, animal studies using resting-state fMRI inevitably require sedation of animals, which limits exploration of awake resting-state functional connectivity^30^. In contrast, ^18^F-FDG uptake reflects activity in the awake state since animals are sedated only after the completion of ^18^F-FDG distribution during the resting state. Furthermore, regional ^18^F-FDG uptake can be considered a more direct measure of neuronal activity than blood oxygenation level dependent signals in fMRI, as neurons exhibit preferential uptake of glucose in an activity-dependent manner^31^. Although ^18^F-FDG PET images do not provide time series data for the conventional evaluation of functional connectivity, introduction of cross-sectional analysis of ^18^F-FDG PET imaging allows researchers to evaluate between-group differences in both regional activity and connectivity^32^.

We first isolated spatially independent nodes of ^18^F-FDG uptake using cross-sectional independent component analysis (ICA). We then examined univariate changes in the weights (hereafter referred to as the activity of each component) of the independent metabolic spatial components (IC) associated with VPA exposure. Then, we evaluated metabolic connectivity between pairs of nodes based on measures of metabolic activity and explored group differences in metabolic connectivity among components using a group-level partial correlation analysis of IC activity in the rat brain^33,34^.

Because VPA treatment results in varying degrees of behavioral deficits on tests of social interaction, we evaluated whether specific changes in distributed metabolic activity correlate with the magnitude of deficits in social behavior. By doing so, we tried to reveal the neurobiological underpinnings of social functioning. Finally, we explored how sex differences modulate the effects of VPA treatment on social behavior and metabolic connectivity. Although the male predominance in ASD is well known, and VPA affects social behaviors differently by sex^16^, the neurobiological mechanism underlying the male predominance is not yet clear^35^. By analyzing brain metabolism and connectivity between metabolic nodes using ^18^F-FDG PET, we thus aimed to reveal a link between reduced performance on the social interaction test and the sex-specific neurobiological alterations observed in the VPA-induced rat model of ASD.

## Results

### Three-chamber social approach test

**Figure 1:**
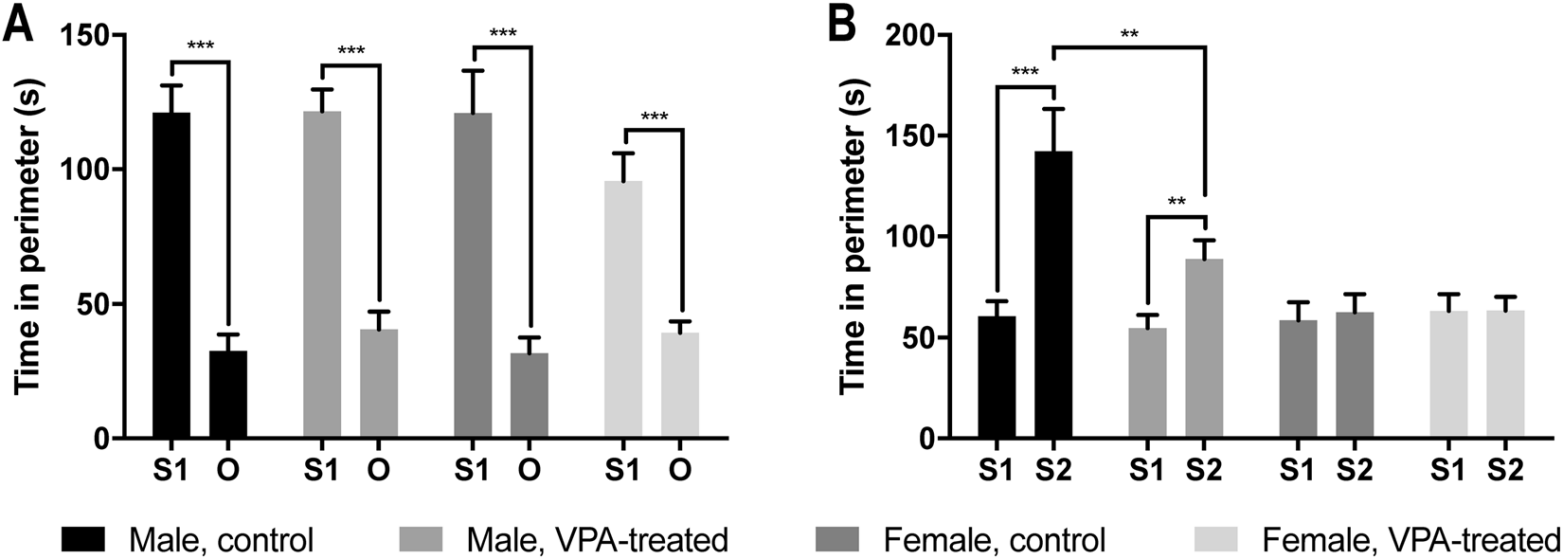
Sociability and social novelty preference test. (**A**) Sociability test. No group difference was found. (**B**) Social novelty preference test. VPA-exposed male rats spent less time with S2 than did control male rats. Data are represented as mean ± SEM. VPA, valproic acid; O, object; S1, stranger 1; S2, stranger 2; SEM, standard error of the mean. **P < 0.01, ***P < 0.001.

In the sociability test, a two-way analysis of variance (ANOVA) indicated no significant main effects of VPA treatment or sex on the time spent with the first stranger (S1) (VPA treatment, F_1,73_ = 1.20, P = 0.28; sex, F_1,73_ = 1.33, P = 0.26) or an empty wire cage presented as a novel object (O) (VPA treatment, F_1,73_ = 1.78, P = 0.19; sex, F_1,73_ = 0.03, P = 0.86). All rats preferred exploring S1 to exploring O, irrespective of VPA treatment or sex: time spent with S1, control male, n = 17, 121.02 ± 10.12 seconds; VPA-treated male, n = 22, 121.60 ± 8.05 seconds; control female, n = 19, 120.96 ± 15.69 seconds; and VPA-treated female, n = 19, 95.62 ± 10.27 seconds; time spent with O, control male, 32.50 ± 6.13 seconds; VPA-treated male, 40.60 ± 6.58 seconds; control female, 31.71 ± 5.84 seconds; and VPA-treated female, 39.29 ± 4.27 seconds. When we calculated the time difference between S1 and O (calculated as [(time spent with S1 - time spent with O) over (time spent with S1 + time spent with O)]), we found that VPA-treated female rats exhibited significant reduction in time difference than controls (VPA-treated female, 0.40 ± 0.04; control female, 0.60 ± 0.04; P = 0.002) (Fig. 1A).

In the social novelty preference test, a two-way ANOVA revealed significant main effects of VPA treatment (F_1,73_ = 4.76, P = 0.03) and sex (F_1,73_ = 18.76, P < 0.001) with an interaction between these factors (F_1,73_ = 5.01, P = 0.03) on the time spent with the second stranger (S2). VPA-treated male rats (88.70 ± 9.39 seconds) spent less time with S2 than did control male rats (142.24 ± 21.07 seconds) (P = 0.002); yet, both groups of male rats maintained preference for S2 over S1 (control male, 60.60 ± 7.49 seconds; and VPA-treated male, 54.67 ± 6.56 seconds), regardless of treatment. Whereas, no female rats showed preference for S2 over S1 irrespective of VPA treatment: time spent with S2, control female, 62.65 ± 8.88 seconds; and VPA-treated female, 63.34 ± 6.86 seconds; time spent with S1, control female, 58.66 ± 8.88 seconds; and VPA-treated female, 63.17 ± 8.39 seconds (Fig. 1B).

### Activity analysis of independent components

**Figure 2:**
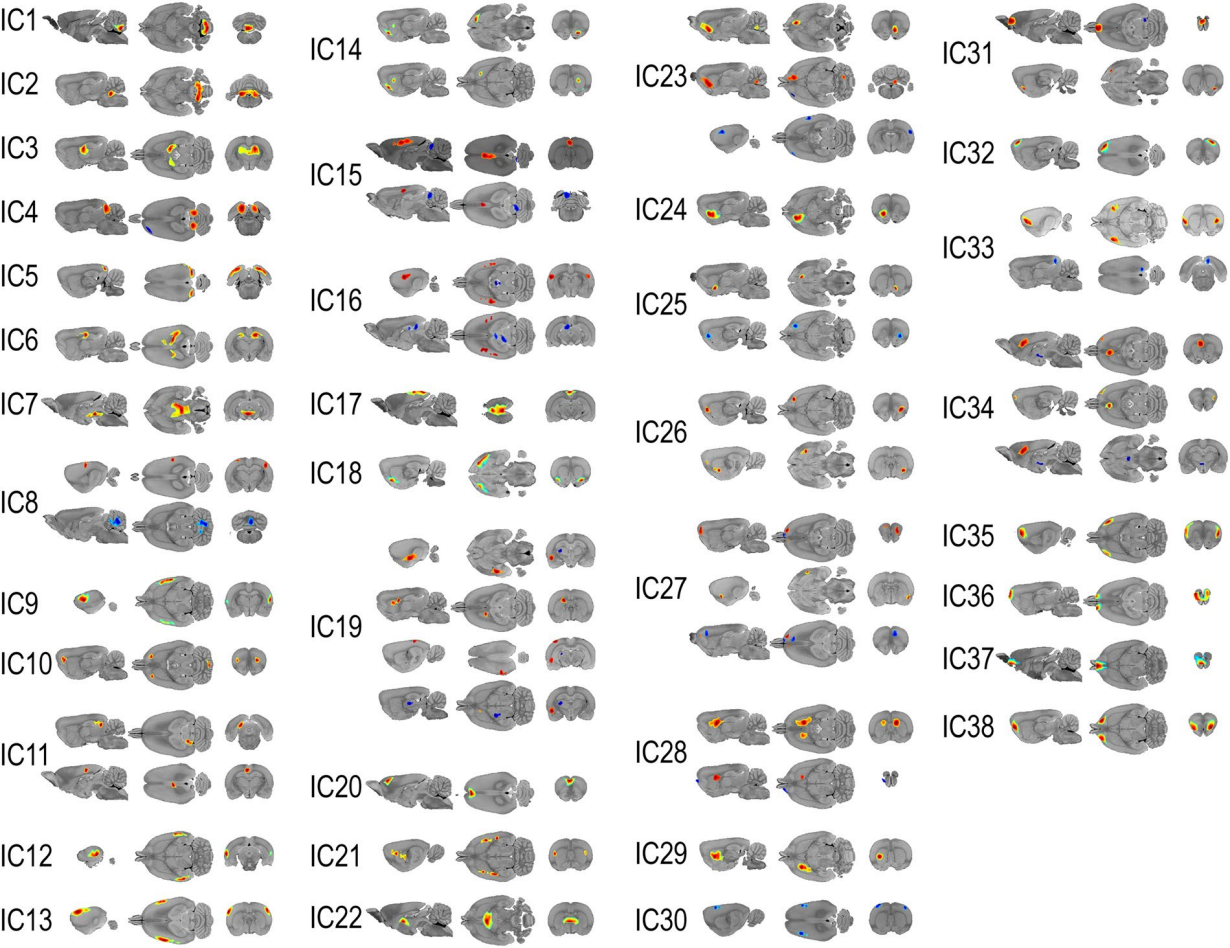
Spatial distribution of independent components. Each component is shown in sagittal (left), transverse (middle), and coronal (right) planes. In each component, regions with intensities greater than two standard deviations from the mean are highlighted. Red indicates regions with positive values, and blue indicates regions with negative values. Independent components included in the connectivity estimation are listed in the following. IC1, positive: cerebellum, both; IC2, positive: medulla, both; IC3, positive: thalamus, both; IC4, positive: inferior colliculus, both; IC4, negative: motor cortex, left, and orbitofrontal cortex, left; IC5, positive: visual cortex, both; IC6, positive: anterodorsal hippocampus, both; IC7, positive: ventral tegmental area, both, and hypothalamus, both; IC8, positive: somatosensory cortex, both; IC8, negative: cerebellum, both; IC9, positive: somatosensory cortex, both; IC10, positive: orbitofrontal cortex, both, and cerebellum, both; IC11, positive: superior colliculus and inferior colliculus, left, and retrosplenial cortex, both; IC12, positive: auditory cortex, both; IC13, positive: somatosensory cortex, both; IC14, positive: olfactory bulb, right, and caudoputamen, right; IC15, positive: cingulate cortex, both; IC15, negative: cerebellum, left; IC16, positive: somatosensory cortex, both; IC16, negative: superior colliculus, left; IC17, positive: retrosplenial cortex, both; IC18, positive: entorhinal cortex, both; IC19, positive: amygdala, left, caudoputamen, left, and visual cortex, left; IC19, negative: thalamus, left; IC20, positive: cingulate cortex, both; IC21, positive: caudoputamen, both; IC22, positive: thalamus, both; IC23, positive: nucleus accumbens, right, and cerebellum, right; IC23, negative: somatosensory cortex, right, orbitofrontal cortex, left, and insular cortex, left; IC24, positive: nucleus accumbens, left; IC25, positive: nucleus accumbens, right; IC25, negative: orbitofrontal cortex, right; IC26, positive: orbitofrontal cortex, right, and caudoputamen, right; IC27, positive: orbitofrontal cortex, both, and entorhinal cortex, right; IC27, negative: olfactory bulb, right, and medial prefrontal cortex, right; IC28, positive: caudoputamen, both; IC28, negative: olfactory bulb, left; IC29, positive: caudoputamen, left; IC30, negative: somatosensory cortex, both; IC31, positive: olfactory bulb, both, and entorhinal cortex, right; IC31, negative: inferior colliculus, right; IC32, positive: motor cortex, right; IC33, positive: insular cortex, both; IC33, negative: retrosplenial cortex, right; IC34, positive: medial prefrontal cortex, left, cingulate cortex, left, and orbitofrontal cortex, right; IC34, negative: hypothalamus, left; IC35, positive: insular cortex, both, and motor cortex, both; IC36, positive: olfactory bulb, both; IC37, positive: olfactory bulb, both; IC38, positive: olfactory bulb, both. IC, independent component.

ICA revealed 38 components (Fig. 2). A two-way ANOVA of IC weights (metabolic activity) showed a significant main effect for VPA treatment in three independent components: the left caudoputamen (IC29, F_1,73_ = 14.92, P < 0.001), olfactory bulb (IC37, F_1,73_ = 14.13, P < 0.001), and thalamus (IC3, F_1,73_ = 10.87, P = 0.002). Significance sex differences were seen in six independent components: the visual cortex (IC5, F_1,73_ = 19.40, P < 0.001), medulla (IC2, F_1,73_ = 14.48, P < 0.001), inferior colliculus, left motor cortex, and left orbitofrontal cortex (IC4, F_1,73_ = 12.17, P < 0.001), somatosensory cortex and cerebellum (IC8, F_1,73_ = 9.02, P = 0.004), left superior and inferior colliculi, and retrosplenial cortex (IC11, F_1,73_ = 8.35, P = 0.005), and somatosensory cortex (IC13, F_1,73_ = 8.17, P = 0.006). No significant interaction between the influence of VPA treatment and sex was found on metabolic activity.

**Figure 3:**
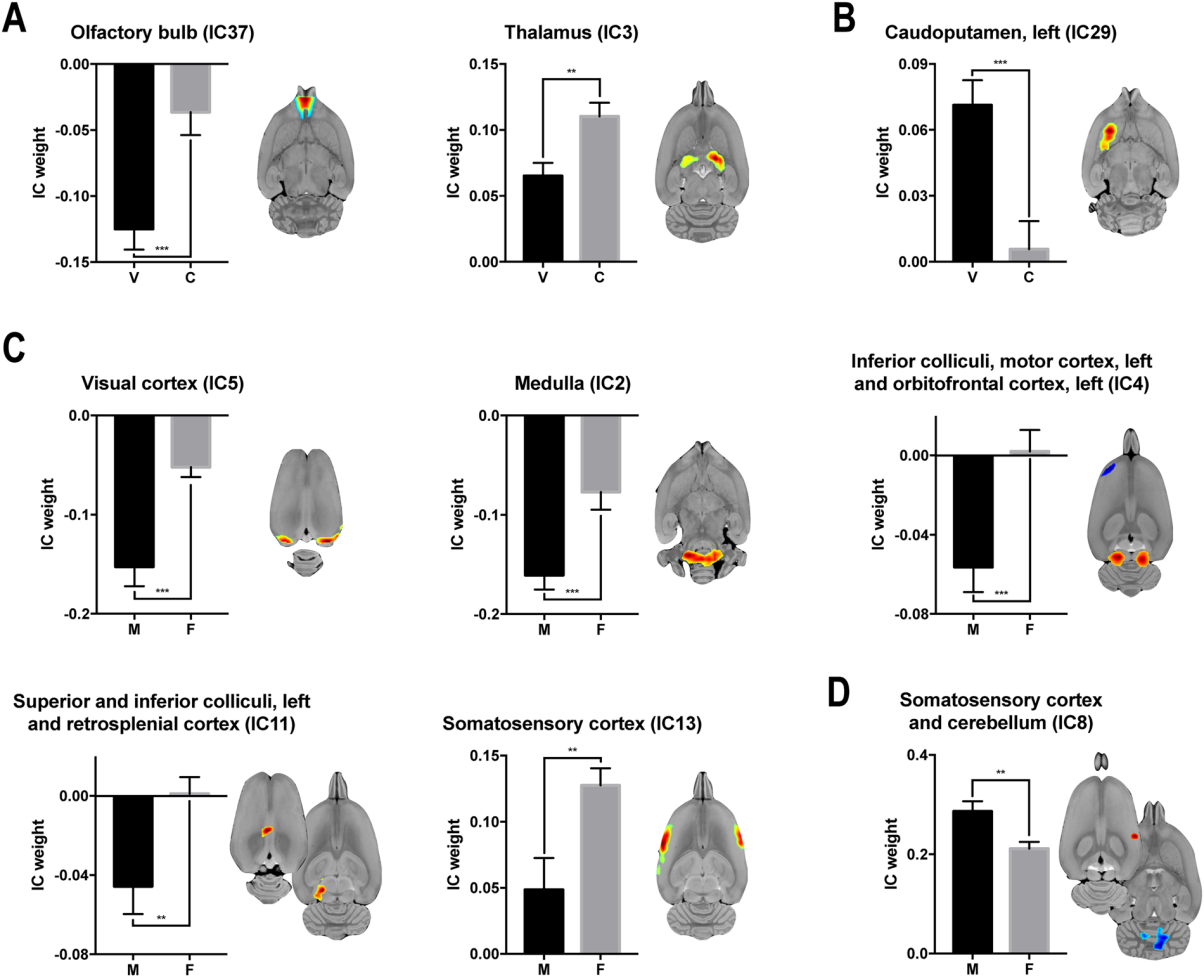
Activity changes induced by prenatal VPA exposure and the interaction with sex. (**A**) Regions with decreased activity in the group exposed to prenatal VPA. (**B**) Regions with increased activity in the group exposed to prenatal VPA. (**C**) Regions where activity decreased more in males than in females. (**D**) Regions where activity increased more in males than in females. Each component is shown in transverse plane. Red indicates regions with positive values, and blue indicates regions with negative values. VPA, valproic acid; IC, independent component; V, valproic-acid treated; C, control; M, male; F, female. **P < 0.01, ***P < 0.001.

FDR-corrected *post hoc* permutation t-tests revealed that the metabolic activity in the independent components encompassing the olfactory bulb (IC37, P < 0.001), and thalamus (IC3, P = 0.001) was significantly decreased in subjects with VPA treatment compared to controls (Fig. 3A); whereas, the metabolic activity in the left caudoputamen (IC29, P < 0.001) was significantly increased in subjects with VPA treatment compared to controls (Fig. 3B). The metabolic activity in the visual cortex (IC5, P < 0.001), medulla (IC2, P < 0.001), inferior colliculus (IC4, P < 0.001), cerebellum (IC8, P = 0.001), left superior and inferior colliculi, and retrosplenial cortex (IC11, P = 0.002), and somatosensory cortex (IC13, P = 0.003) was significantly decreased in males compared to females (Fig. 3C). In contrast, the metabolic activity in the left motor cortex, and left orbitofrontal cortex (IC4, P < 0.001), and somatosensory cortex (IC8, P = 0.001) was significantly increased in males compared to females (Fig. 3D, Table 1).

**Table 1:** Significant effects of prenatal VPA exposure and sex on the activity of independent components in rats.

IC	Activity		P
*Decreased by VPA treatment*	*VPA-treated*	*Control*	
Olfactory bulb (IC37)	−0.12 ± 0.02	−0.04 ± 0.02	<0.001
Thalamus (IC3)	0.07 ± 0.01	0.11 ± 0.01	0.002
*Increased by VPA treatment*	*VPA-treated*	*Control*	
Caudoputamen, left (IC29)	0.07 ± 0.01	0.01 ± 0.01	<0.001
*Decreased in males*	*Male*	*Female*	
Visual cortex (IC5)	−0.15 ± 0.02	−0.05 ± 0.01	<0.001
Medulla (IC2)	−0.16 ± 0.01	−0.08 ± 0.02	<0.001
Inferior colliculus (IC4)	−0.06 ± 0.01	0.00 ± 0.01	<0.001
Cerebellum (IC8)	−0.29 ± 0.02	−0.21 ± 0.01	0.002
Superior colliculus and inferior colliculus, left and retrosplenial cortex (IC11)	−0.05 ± 0.01	0.00 ± 0.01	0.005
Somatosensory cortex (IC13)	0.05 ± 0.02	0.13 ± 0.01	0.004
*Increased in males*	*Male*	*Female*	
Motor cortex, left and orbitofrontal cortex, left (IC4)	0.06 ± 0.01	0.00 ± 0.01	<0.001
Somatosensory cortex (IC8)	0.29 ± 0.02	0.21 ± 0.01	0.002

A FDR ≤ 0.05 was considered as statistically significant. VPA, valproic acid; IC. Independent component.

### Connectivity estimated by sparse inverse covariance estimation

**Figure 4:**
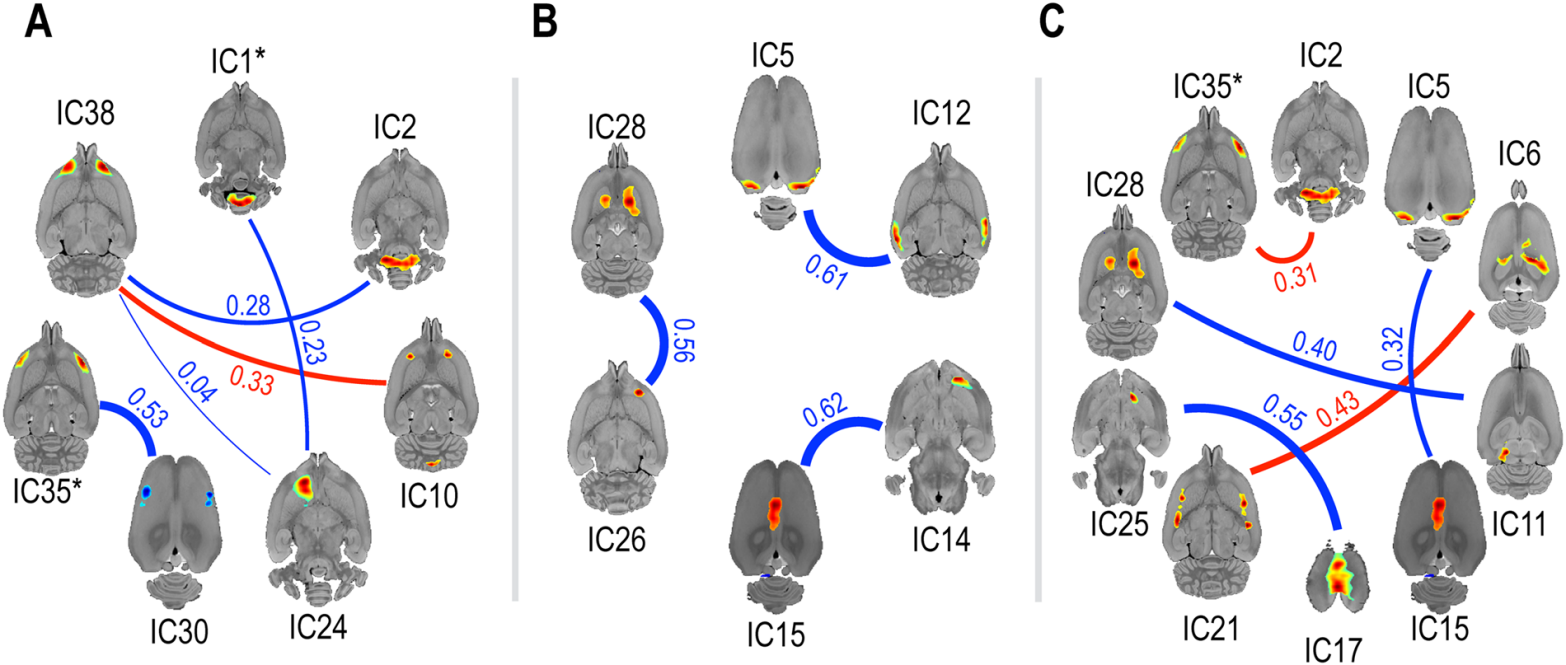
Connectivity changes induced by prenatal VPA exposure and the interaction with sex, as estimated by sparse inverse covariance estimation. (**A**) The main effect of VPA exposure: the blue line indicates the connections weakened by VPA treatment, while the red line indicates the connections strengthened by VPA treatment. (**B**) The main effect of sex: the blue line indicates connections that were weaker in males than females. (**C**) The interaction effect between VPA exposure and sex: the blue line indicates areas with less pronounced connectivity increases in males when compared to females following VPA exposure, while the red line indicates more pronounced increases in connectivity in males when compared to females following VPA treatment. The activity in the IC1* (the cerebellum) and IC35* (the insula) were correlated with autistic-like behavior in Fig. 5. Each component is shown in transverse plane. Line thickness and the corresponding number indicate the relative strength ($\tilde{{\rm{\theta }}}$, estimated partial correlation coefficient) of the connections. Only the connections with a FDR ≤ 0.05 were shown. VPA, valproic acid; IC, independent component.

To examine the possible influence of VPA exposure on metabolic connectivity, we estimated partial-correlation matrices of IC weights using sparse inverse covariance estimation (SICE). Connectivity between four pairs of components, or ICA networks, were significantly weaker in rats exposed to VPA treatment: olfactory bulb (IC38) and left nucleus accumbens (IC24) (VPA-treated, $\tilde{{\rm{\theta }}}$ = 0; control, $\tilde{{\rm{\theta }}}$ = 0.04; P < 0.001); somatosensory cortex (IC30) and insular cortex, and motor cortex (IC35) (VPA-treated, $\tilde{{\rm{\theta }}}$ = 0; control, $\tilde{{\rm{\theta }}}$ = 0.53; P < 0.001); olfactory bulb (IC38) and medulla (IC2) (VPA-treated, $\tilde{{\rm{\theta }}}$ = 0; control, $\tilde{{\rm{\theta }}}$ = 0.28; P = 0.003); and cerebellum (IC1) and left nucleus accumbens (IC24) (VPA-treated, $\tilde{{\rm{\theta }}}$ = 0; control, $\tilde{{\rm{\theta }}}$ = 0.23; P = 0.005). Connectivity between one pair of components, or ICA network, was significantly stronger in rats exposed to VPA treatment: olfactory bulb (IC38) and orbitofrontal cortex, and cerebellum (IC10) (VPA-treated, $\tilde{{\rm{\theta }}}$ = 0.33; control, $\tilde{{\rm{\theta }}}$ = 0; P < 0.001) (Fig. 4A).

We also assessed sex-specific changes in brain connectivity. Three ICA networks, or component pairs, were significantly weaker in males compared to females: visual cortex (IC5) and auditory cortex (IC12) (males, $\tilde{{\rm{\theta }}}$ = 0; females, $\tilde{{\rm{\theta }}}$ = 0.61; P < 0.001); right orbitofrontal cortex, and right caudoputamen (IC26) and caudoputamen, and left olfactory bulb (IC28) (males, $\tilde{{\rm{\theta }}}$ = 0; females, $\tilde{{\rm{\theta }}}$ = 0.56; P = 0.003); and right olfactory bulb, and right caudoputamen (IC14) and cingulate cortex, and left cerebellum (IC15) (males, $\tilde{{\rm{\theta }}}$ = 0; females, $\tilde{{\rm{\theta }}}$ = 0.62; P = 0.004). No ICA network was significantly stronger in males compared to females (Fig. 4B).

The SICE analysis also revealed significant interactions between VPA treatment and sex on brain connectivity. In three ICA networks, connections were less increased in males than in females by VPA treatment: retrosplenial cortex (IC17) and right nucleus accumbens, and right orbitofrontal cortex (IC25) (males, $\tilde{{\rm{\theta }}}$ = 0; females, $\tilde{{\rm{\theta }}}$ = 0.55; P = 0.003); visual cortex (IC5) and cingulate cortex, and left cerebellum (IC15) (males, $\tilde{{\rm{\theta }}}$ = 0; females, $\tilde{{\rm{\theta }}}$ = 0.32; P = 0.005); and left superior and inferior colliculi, and retrosplenial cortex (IC11) and caudoputamen, and left olfactory bulb (IC28) (males, $\tilde{{\rm{\theta }}}$ = 0; females, $\tilde{{\rm{\theta }}}$ = 0.40; P = 0.01). In two ICA networks, connections were more increased in males than in females by VPA treatment: anterodorsal hippocampus (IC6) and caudoputamen (IC21) (males, $\tilde{{\rm{\theta }}}$ = 0.43; females, $\tilde{{\rm{\theta }}}$ = 0; P < 0.001); and medulla (IC2) and insular cortex, and motor cortex (IC35) (males, $\tilde{{\rm{\theta }}}$ = 0.31; females, $\tilde{{\rm{\theta }}}$ = 0; P < 0.001) (Fig. 4C, Table 2).

**Table 2:** Effects of prenatal VPA exposure and sex on brain connectivity in rats estimated by sparse inverse covariance estimation.

Connectivity	Strength		P
*Decreased by VPA treatment*	*VPA-treated*	*Control*	
Olfactory bulb (IC38) ↔ nucleus accumbens, left (IC24)	0	0.04	<0.001
Somatosensory cortex (IC30) ↔ insular cortex, and motor cortex (IC35)	0	0.53	<0.001
Olfactory bulb (IC38) ↔ medulla (IC2)	0	0.28	0.003
Cerebellum (IC1) ↔ nucleus accumbens, left (IC24)	0	0.23	0.005
*Increased by VPA treatment*	*VPA-treated*	*Control*	
Olfactory bulb (IC38) ↔ orbitofrontal cortex, and cerebellum (IC10)	0.33	0	<0.001
*Decreased in males*	*Male*	*Female*	
Visual cortex (IC5) ↔ auditory cortex (IC12)	0	0.61	<0.001
Orbitofrontal cortex, right, and caudoputamen, right (IC26) ↔ caudoputamen, and olfactory bulb, left (IC28)	0	0.56	0.003
Olfactory bulb, right, and caudoputamen, right (IC14) ↔ cingulate cortex, and cerebellum, left (IC15)	0	0.62	0.004
*More increased in males than in females by VPA treatment*: [(*male* − *female)* × *(VPA-treated - control*)]	*Male*	*Female*	
Anterodorsal hippocampus (IC6) ↔ caudoputamen (IC21)	0.43	0	<0.001
Medulla (IC2) ↔ insular cortex, and motor cortex (IC35)	0.31	0	<0.001
*Less increased in males than in females by VPA treatment*: [(*female* − *male)* × *(VPA-treated - control*)]	*Male*	*Female*	
Retrosplenial cortex (IC17) ↔ nucleus accumbens, right, and orbitofrontal cortex, right (IC25)	0	0.55	0.003
Visual cortex (IC5) ↔ cingulate cortex, and cerebellum, left (IC15)	0	0.32	0.005
Superior and inferior colliculus, left, and retrosplenial cortex (IC11) ↔ caudoputamen, and olfactory bulb, left (IC28)	0	0.40	0.01

A FDR ≤0.05 was considered as statistically significant. VPA, valproic acid; IC. Independent component.

As a reference, the same evaluations were conducted with Pearson correlation of all pairs of IC weights. Permutation testing of Pearson correlation matrices revealed that no connection was significantly affected by VPA treatment or sex at a FDR threshold of 0.05. There was no significant interaction between VPA treatment and sex on connectivity as assessed by Pearson’s correlation.

### Sparse canonical correlation analysis

**Figure 5:**
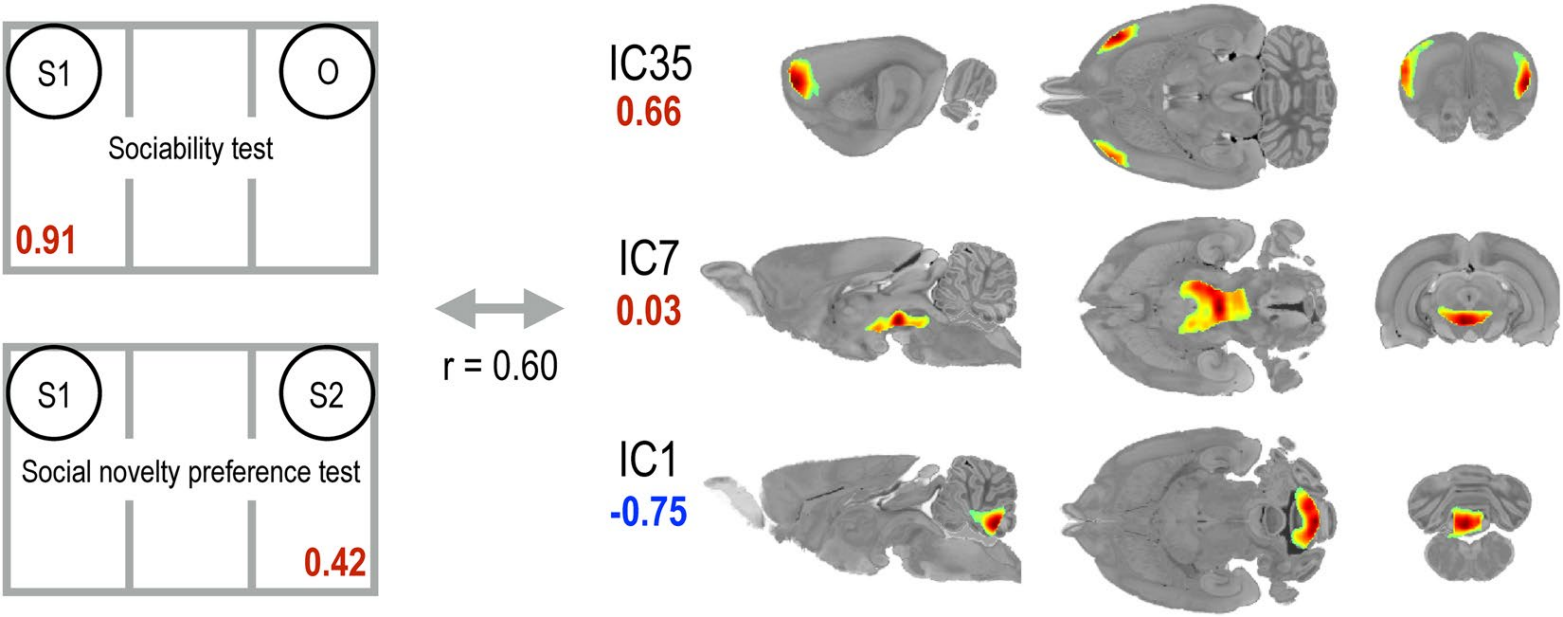
Spatial patterns associated with normal social behavior revealed by sparse canonical correlation analysis. Normal social behavior was positively correlated with activity in the insular cortex and motor cortex (IC35), as well as that in the ventral tegmental area and hypothalamus (IC7), and negatively correlated with activity in the cerebellum (IC1). The relative contribution of each behavior or IC is shown in numbers. The number in red indicates positive correlation, and the number in blue indicates negative correlation. Each component is shown in sagittal (left), transverse (middle), and coronal (right) planes. IC, independent component.

A significant canonical covariate that modeled the relationship between behavioral parameters and sparse metabolic networks emerged from the sparse canonical correlation analysis. The behavioral parameters favoring social interactions (0.91 × [time spent with S1 in sociability test] + 0.42 × [time spent with S2 in social novelty preference test]) were significantly correlated with a specific combination of three distributed metabolic activity patterns (r = 0.60, P < 0.001). Normal social behavior was positively correlated with activity in the insular cortex and motor cortex (IC35) (+0.66), ventral tegmental area and hypothalamus (IC7) (+0.03), and was negatively correlated with activity in the cerebellum (IC1) (−0.75) (Fig. 5).

**Figure 6:**
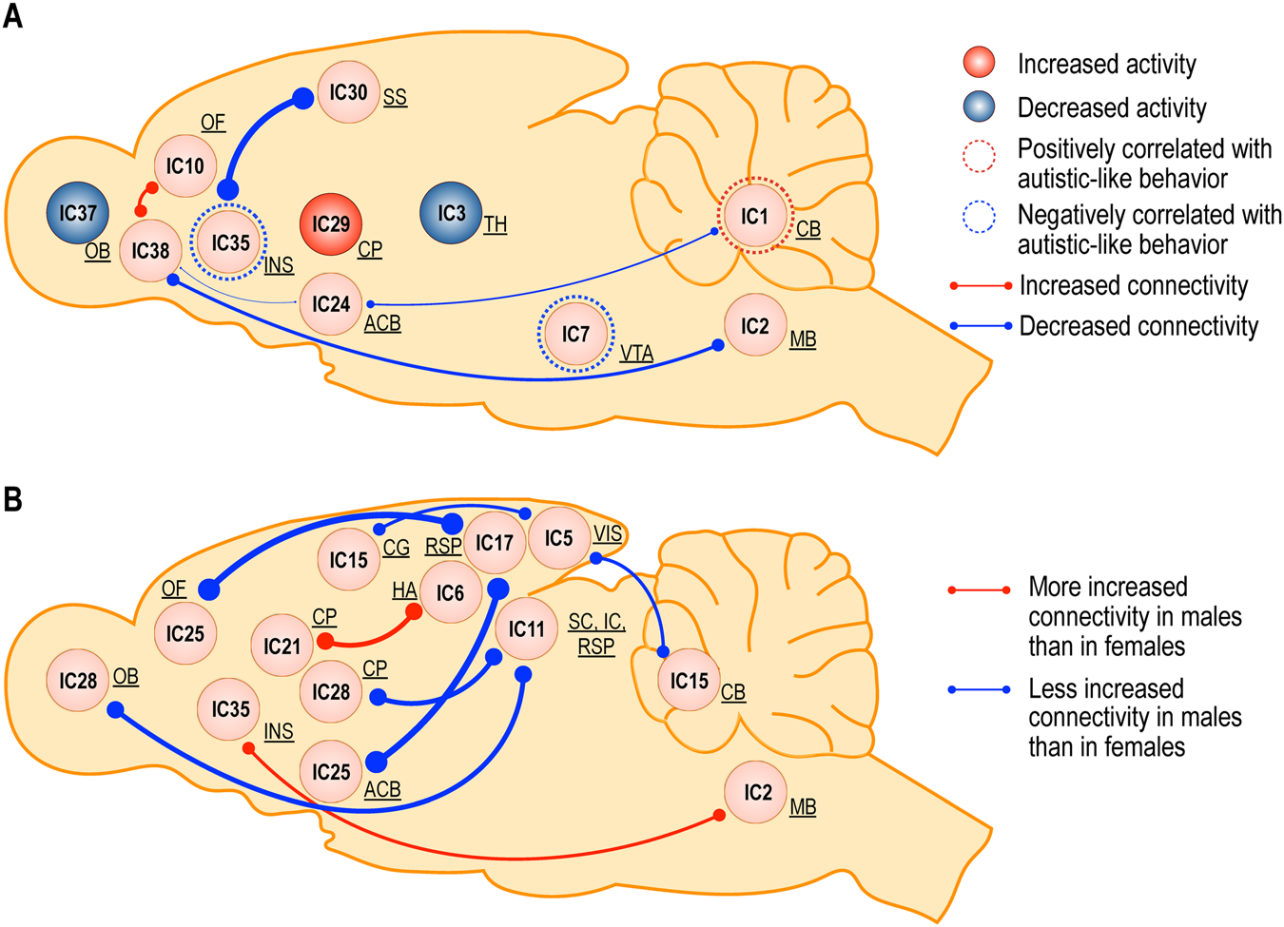
Activity and connectivity changes induced by prenatal VPA exposure, and brain regions associated with normal social behavior (**A**) and the effect of VPA exposure on sex (**B**). (**A**) The main effects of VPA exposure and the results from sparse canonical correlation analysis are shown. The blue circle indicates the regions with diminished activity in the group exposed to prenatal VPA, while the red circle indicates the regions with increased activity in the VPA exposed group. The blue line indicates the connections weakened in the group exposed to VPA, while the red line indicates the connections strengthened in the VPA exposed group. Line thickness indicates the relative strength of the connections. The blue dashed circle indicates the regions positively correlated with normal social behavior, while the red dashed circle indicates the regions negatively correlated with normal social behavior. (**B**) Interaction on connectivity between VPA exposure and sex are displayed. The red line indicates the connections more strengthened in males than in females when exposed to VPA, while the blue line indicates the connections less increased in males than in females by VPA exposure. VPA, valproic acid; IC, independent component; NAc, nucleus accumbens; CB, cerebellum; CG, cingulate cortex; CP, caudoputamen; HA, anterodorsal hippocampus; HY, hypothalamus; IC, inferior colliculus; INS, insular cortex; MB, midbrain; OB, olfactory bulb; OF, orbitofrontal cortex; RSP, retrosplenial cortex; SC, superior colliculus; SS, somatosensory cortex; TH, thalamus; VIS, visual cortex; VTA, ventral tegmental area.

Figure 6 summarizes the main findings from the current study. (A) VPA exposure *in utero* is associated with 1) decreased metabolism in the olfactory bulb (IC37) and thalamus (IC3), and increased metabolic activity in the left caudoputamen (IC29); 2) diminished connectivity between the insular (IC35) and somatosensory cortex (IC30), between the olfactory bulb (IC38) and both the nucleus accumbens (IC24) and medulla (IC2), and between the cerebellum (IC1) and nucleus accumbens (IC24); and, 3) enhanced connectivity between the orbitofrontal cortex (IC10) and olfactory bulb (IC38). Activity in the insular cortex (IC35), hypothalamus, and ventral tegmental area (IC7) positively correlated with measures of normal social behavior; whereas, diminished activity in the cerebellum (IC1) correlated with normal social behavior. (B) Significant interactions between VPA exposure and sex only emerged by examining metabolic connectivity, rather than metabolic activity. VPA exposure increased the connections between the components associated with the retrosplenial cortex (IC11 and 17), and both the orbitofrontal cortex (IC25a) and nucleus accumbens (IC25b), and olfactory bulb (IC28a) and caudoputamen (IC28b) less in males compared to females. VPA exposure also increased the connectivity between the visual cortex (IC5), and the component that includes both the cingulate cortex (IC15a) and cerebellum (IC15b) to a lesser extent in males than in females. Whereas, VPA exposure was associated with enhanced connectivity between the components encompassing the anterodorsal hippocampus (IC6) and caudoputamen (IC21) to a greater extent in males compared to females. VPA exposure also increased the connections between the medulla (IC2) and insular cortex (IC35) to a greater extent in males than in females.

## Discussion

The pathophysiology of ASD, a behaviorally defined heterogeneous disorder, remains elusive. Many neuroimaging efforts aimed at identifying commonalities in patients with ASD are hampered by the diverse manifestations of the disease. Thus, we focused on a homogeneous type of ASD induced by prenatal exposure to VPA. We hypothesized that, under the controlled condition of VPA exposure, alterations in brain metabolism and networks of metabolic connectivity would provide insight into the neurobiological basis of ASD. We also explored sex-specific changes both in behavior and in the connectivity between metabolic nodes following VPA treatment.

### VPA treatment results in sex-specific impairments in social interaction

Similar to other studies that have examined autistic-like behavior induced by VPA exposure, our findings are sex specific^16^. When we used sniffing time as a measure for social interaction (sociability and social novelty preference)^36^, male VPA rats exhibited impaired preference for a novel rat (S2) compared to a familiar rat (S1) relative to control rats. In contrast to male VPA rats, female VPA rats did not show any significant difference in the social novelty preference test, but they showed preference to an empty wire cage (O) compared to control rats in the sociability test.

Contrary to the general population, where ASD is four to five times more common in males than females, studies on the prevalence of ASD in children prenatally exposed to VPA have reported a more even male to female ratio^12^. A later study also confirmed that the sex ratio (males to females) of ASD differed between the population exposed to valproate (2.4 males to 1 female) as compared to those not exposed to valproate (4.4 males to 1 female), suggesting that females may share a more equivalent vulnerability to this particular etiology^13^. Similarly, in rodents, prenatal exposure to VPA induces the autistic-like phenotype with a similar incidence in both sexes^37,38^. However, subtle differences between the sexes were observed in the behavioral and cellular alterations. Although female mice exposed to VPA on day 12.5 of gestation demonstrated similar behavioral changes to males on measures of anxiety, measure of sociability differed between the sexes. Both females and males exposed to VPA spent more time in the periphery of the field in the open field test and preferred the enclosed arms in the elevated plus maze test compared to non-exposed age matched controls. However, unlike the VPA exposed males that generally ignored an age-matched stranger mouse, VPA exposed females spent more time sniffing the stranger mouse^37^. The anatomical distribution of CNS cell loss differed between the sexes as well. Although Nissl-positive cell loss in the prefrontal cortex was observed in both males and females exposed to VPA, cell loss in the somatosensory cortex was only detected in male rats^38^. Another study also identified that, although both male and female mice exposed to VPA exhibited increased repetitive/stereotypic-like activity and had a decreased IFN-γ/IL-10 ratio, only the male mice also displayed a lower sensitivity to pain and a decreased level of social interactions^16^. In line with these results, we also observed less pronounced behavioral alterations in VPA-exposed female rats, as the two groups of female rats did not behave significantly differently in the social novelty preference test. As social interaction is mediated by olfaction^39^, an olfactory deficit might have affected the results of the social interaction test^15,40^. However, both groups of female rats displayed significant preference for stranger 1 over a novel object, suggesting that the female rats maintained their ability to recognize a conspecific and therefore that an olfactory deficit does not explain their indifference to stranger 2 versus stranger 1. These sex-specific behavioral findings are consistent with those of previous reports suggesting that the core symptoms of ASD manifest differently in males as compared to females^41^. Although precisely how VPA induces sex-specific behavioral changes remains unclear, these changes may be associated with sex-dependent alterations in metabolic connectivity, as discussed in the following section.

### VPA treatment changes metabolic activity and connectivity

As mentioned before, neuroimaging studies in ASD report diverse findings and definitive neural correlates of ASD have not yet been identified. Among the few studies that have investigated resting-state brain glucose metabolism in humans with ASD, no consensus has emerged. Indeed, some studies have observed no differences between patients with ASD and controls^42^, while others have reported fewer positive correlations between frontal and parietal regions^43^ or widespread hypermetabolism in individuals with ASD^44^. These studies are mostly based on regional metabolic differences, with the exception of one exploration of resting-state metabolic connectivity in humans with ASD^43^. That study used correlation analysis to estimate the metabolic connectivity among frontal cortices, parietal cortices, and subcortical structures, and reported that patients with ASD showed impaired interactions between frontal/parietal regions and the neostriatum and thalamus.

In this study, we used ICA analysis to examine alterations in cross-sectional metabolic networks, which provides a data-driven parcellation of brain regions and their activities (represented in the form of IC weights). This method is widely accepted in neuroimaging communities since it represents functional or biological clusters better than structurally defined region of interests^45^.

ICA analysis revealed that VPA treatment decreased activity (IC weight) in the olfactory bulb (IC37) and thalamus (IC3), and increased activity in the left caudoputamen (IC29) in both male and female rats. Evidence from clinical and preclinical studies implicate these brain regions in the pathophysiology of ASD. The details of how our findings might relate to previous work is addressed in the following section.

Olfaction supports many aspects of social behavior in rats, from maternal care, to reproduction. Diminished function of the olfactory system has been implicated in both rats and humans with ASD. Previous research has reported that the olfaction induced nest-seeking response is delayed in male VPA rats^15^. In humans, children with ASD show little preference for pleasant over unpleasant odors, in contrast to that observed for typically-developing children^46^. Increasing evidence supports the idea that social chemo-signaling is an important mediator of social interaction in both humans and rodents, as olfaction fundamentally influences social behaviors^39,47^.

Longitudinal studies have shown that an increase in the growth rate of the caudate nucleus in the striatum is correlated with repetitive behavior in humans with ASD^48,49^. Right caudate volume has also been implicated in repetitive behaviors in ASD^50^. The increased activity observed in the caudoputamen of VPA rats in the present study is consistent with these observations.

The thalamus and connectivity between the thalamus and other brain regions have also been implicated in ASD. Effective thalamocortical connectivity^51^, thalamocortical interactions during auditory processing^52^, and thalamus mediated sensory processing^53^ have all been associated with ASD.

VPA treatment affected not only the regional activity but also connectivity between regions. Although overlap between activity and connectivity changes due to VPA treatment occurred, regions in which alterations in connectivity were observed were not identical to those in which alterations in activity were observed. VPA treatment weakened connectivity between regions involved in social recognition and reward (olfactory bulb (IC38) and in the nucleus accumbens (IC24)), as well as that involved in salience processing and interoception (somatosensory cortex (IC30) and in the insular cortex (IC35)). The salience network processes multiple stimuli perceived by the brain and presumably determines the relevancy of a stimulus in order to respond with an appropriate display of behaviors. The insular cortex plays a central role in salience processing^54^ and previous research has indicated that the anterior part of the insular cortex interprets the state of the organism by integrating external sensory cues with internal bodily sensations^55,56^. The insular cortex has extensive structural connections with the amygdala, orbitofrontal cortex, olfactory cortex, anterior cingulate cortex, and superior temporal sulcus^57,58^. In particular, the activity of the insular cortex, often together with activity in the amygdala, provides interoceptive awareness, as well as bodily perceptions of external stimuli^59,60^. Impaired interoception may be an underlying deficit that precedes other manifestation of ASD in humans, such as theory of mind and social communication^61^.

Another subcortical node of the salience network is the nucleus accumbens/ventral tegmental area, which plays an important role in reward prediction^62^. The nucleus accumbens plays a crucial role in processing social information via dopamine reward circuitry, and diminished saliency of social stimuli is associated with ASD in humans^63^ as well as abnormal social information processing^64^. Previous studies have revealed that altered salience network connectivity or reduced salience network integrity is associated with the core symptoms of ASD^65,66^. Impaired salience processing of social and interoceptive stimuli may serve as a pathognomonic sign in VPA-induced rat model of ASD.

Connectivity among the olfactory bulb (IC38), orbitofrontal cortex, and cerebellum (IC10) was paradoxically strengthened by VPA treatment. These regions have also been implicated in the pathophysiology of ASD. A previous report suggested that abnormal face detection by the superior colliculus and impaired emotion processing by the orbitofrontal cortex also contribute to abnormal social processing in patients with ASD^67^.

### Variations in the autistic-like behavior score were associated with distributed metabolic activity patterns

Not all animals exposed to VPA treatment *in utero* exhibited autistic-like behaviors. The heterogeneity of both behavioral manifestations and changes in metabolic distribution requires an integrated analytic approach that combines behavioral and metabolic measures into a single, comprehensive multivariate analysis. Thus, we used sparse canonical correlation analysis (SCCA) to link behavioral measures with specific patterns of metabolic activity. SCCA maximizes the correlation between behavioral and metabolic variables while minimizing the number of variables used in both sets^68^.

SCCA identified that normal social interaction was correlated with specific distributed brain activity patterns: a positive correlation existed between normal social behavior and increased metabolic activity in the salience network and related structures (IC35 and IC7), and negative correlation was seen between normal social behavior and increased metabolic activity in the cerebellum component (IC1).

As discussed earlier, the salience network (IC35) attends to behaviorally relevant stimuli and initiates appropriate responses to these stimuli^69^. Consistent with the connectivity changes in the salience network, this network is highly associated with the degree of social interaction in the rats. A previous report indicated that, in children with ASD, salience-network connectivity was linked with the severity of symptoms^66^. Thus, alterations in the connectivity of the salience network and associated areas may serve as possible neural substrates, reflective of social behavioral symptom severity in ASD.

Increasing evidence supports the notion that the cerebellar region corresponding to IC1 not only mediates motor function but also influences social processing^70^. In humans with ASD, cerebellar abnormalities have been recognized early in life, as core ASD deficits appear, especially in the vermis, an area further correlated with social impairment^71^. Our results suggest that combined abnormalities in these regions contribute to the development of autistic-like behavior in VPA rats.

### VPA treatment affects metabolic connectivity in sex-specific way

While no interaction was identified between the sex of the animal and metabolic activity, we observed significant interactions between VPA treatment and sex in metabolic connectivity, similar to the interactions observed for behavioral measures. Although ASD is known to affect males and females differently, little is known regarding sex-based alterations in brain connectivity. Holt, *et al*.^72^ found that neurotypical men performed better than men with ASD on a mentalizing task, although no differences were noted between neurotypical women and those with ASD. Both men and women with ASD exhibit reduced neural activation relative to controls when processing social information. Another study revealed that men with ASD exhibited decreased neural activity compared to controls during social information processing, while no differences in neural activity were observed between groups of women^73^. These findings suggest that ASD is characterized by both behavioral and neurobiological differences between men and women.

The present study demonstrates that sex-specific differences in behavior following prenatal exposure to VPA treatment were associated with differences in metabolic connectivity. We also reveal that ASD-related changes in brain connectivity—but not in brain activity—following VPA treatment were substantially different between males and females.

Connections between the visual cortex (IC5) and cerebellum (IC15), and between the retrosplenial cortex (IC11 and 17) and the nucleus accumbens (IC25) and the olfactory bulb (IC28) were preferentially less increased in males exposed to VPA compared to VPA exposed females. Given the retrosplenial’s role in orienting and navigation in rodents, and its primary position in the default mode network in humans, connections between this cortical area and other regions could underlie certain aspects of cognition and self-reflection that contribute to the autistic phenotype^74^.

Significant interactions between VPA treatment and sex were observed between networks that may be responsible for decision-making (hippocampus (IC6) and caudoputamen (IC21)) and autonomic regulation (medulla (IC2) and insular cortex (IC35))^75,76^. The enhanced connectivity between the insula, medulla, and motor cortex may reflect the well-documented autonomic dysfunction reported in ASD and its relation to social functioning^77^. Given the greater severity of the behavioral deficits witnessed in the male rats, it is unclear whether enhanced connectivity represents an attempt to compensate for other areas of diminished activity or connectivity, or an example of the excitatory/inhibitory imbalance discussed below.

### Implications for ASD

Although the mechanism underlying the development of autistic-like behavior induced by VPA remains poorly understood, an increasing number of studies have suggested that embryonic VPA exposure contributes to an imbalance between excitatory and inhibitory neurotransmission^17,78^. VPA exposure has been demonstrated to suppress GABAergic inhibitory neuronal development^79^, and stimulate excitatory, glutamatergic, neuronal expression^78^. However, where this imbalance occurs anatomically in ASD and how such an imbalance causes autistic behavior is not clearly understood. Recently, alterations in excitatory-inhibitory balance have been proposed to underlie aberrant brain connectivity in many developmental disorders, including ASD^80^. The current study, therefore, suggests that distributed metabolic activity and the connectivity changes observed in the VPA exposed rats may link the gap between cellular alterations (excitatory and inhibitory imbalance) and the abnormal behavior typical of ASD.

Our results indicate that interactions among multiple brain regions, particularly within the salience and cerebellar networks, were involved in the development of autistic-like social behavioral deficits in the VPA-exposed rats. The insular cortex and nucleus accumbens/ventral tegmental area constitute the salience network, mediate social reward, and are involved in social cognition^62,81^. The cerebellum has also been implicated in social and emotional processing, language, and cognition^70^. Taken together, these findings suggest that inappropriate responses to social stimuli resulting from deficient salience attribution by the salience network and abnormal social processing in the cerebellum contribute to autistic-like behavior (Fig. 6). Although VPA exposure represents only one possible risk factor for ASD, the VPA rat model may shed light on alterations in the brain connectivity that occur during the development of ASD.

Our VPA-exposed female rats demonstrated less prominent behavioral derangement and associated changes in brain connectivity. These findings suggest that female rats are less vulnerable to VPA exposure, given the protocol followed. Some evidence suggests that the GABAergic system in the female matures more quickly than in males. Chloride homeostasis proceeds through a developmental shift such that GABA transmission switches from excitatory to inhibitory in the brain after birth^82,83^ and this shift appears to occur earlier in females than in males^84,85^. If this sex-specific difference in the rate of maturation also exists in the prenatal period, the female embryos would have reached a greater level of maturity by E12.5, and would therefore be less susceptible to VPA induced alterations at the time of exposure.

Alternatively, the hormonal milieu in the female embryos may protect against the deleterious effects of VPA exposure. Various studies have suggested that testosterone may mediate some of the downstream changes associated with VPA exposure resulting in the autistic phenotype^86^.

The greater magnitude of social behavioral deficits witnessed in the male rats in this study mirrors the increased male prevalence of autism in humans. The current study revealed that these behavior deficits in male rats, due to the effects of VPA treatment, are associated with changes in distributed brain activity patterns and brain connectivity, particularly in the salience and cerebellar networks.

In the present study, inter-liter variation might have confounded the effects of VPA exposure, as the number of included dams is relatively small (n = 4 for each control or VPA-treated)^87^. However, our evaluation of statistical significance with permutation testing minimizes the confound of small litter numbers^88^.

We did not perform additional social interaction tests at 62 weeks of age due to the potential confound of learning with repeated exposure to the testing paradigm. Although the severity of ASD symptoms often improve with time, the diagnosis of ASD remains generally stable^89^. In line with these findings, a single prenatal exposure to VPA in rodents is known to cause lifelong behavioral impairments that represent the core symptoms of ASD^18^.

The distributed brain activity pattern identified by SCCA overlaps with changes in (direct sparse) connectivity by SICE, but not with changes in activity attributed to VPA exposure. In addition, we did not find shared features between changes in brain connectivity and activity. We might not rule out the possibility that our strict statistical criteria may have obscured subtle changes according to previous studies where changes in brain activity and connectivity often accompany one another in patients with ASD^20^. However, the changes in interregional connectivity appear to be a more sensitive indicator of ASD than the alterations in activity.

In conclusion, differences in patterns of connectivity, as assessed by metabolic correlations, may represent neurobiological substrates of autistic-like behavior in the social realm, particularly in males, and may serve as a pathognomonic sign in the VPA rat model of ASD.

## Methods

All animal procedures were performed in accordance with the institutional animal care and use committee guidelines and approved by the Yonsei University College of Medicine Institutional Animal Care and Use Committee.

### Animals

A total of 77 pups (39 males and 38 females) were enrolled in this study. The pups were classified into eight groups based on age at imaging, sex, and treatment: adolescent, male, control (n = 8); adolescent, male, VPA-treated (n = 7); adolescent, female, control (n = 8); adolescent, female, VPA-treated (n = 8); adult, male, control (n = 9); adult, male, VPA-treated (n = 15); adult, female, control (n = 11); and adult, female, VPA-treated (n = 11).

Eight pregnant Sprague-Dawley (SD) female rats (Orient Bio Inc., Gyeonggi-do, South Korea) were randomly assigned to receive a single subcutaneous injection of sodium valproate (Sigma) dissolved in saline (400 mg/kg), or saline alone on embryonic day 12.5 (E12.5), as previously described (Kim *et al*., 2011). Dams were housed individually and were allowed to raise their own litters. Pups were weaned at postnatal day 21 (P21), and were housed with 2-3 rats in same-sex and same-treatment cages. Standard plastic laboratory cages were used with bedding and *ad libitum* access to food and water, handled twice a week. Animals were kept in a 12-hour light-dark schedule with lights on at 8:00 am, in rooms under controlled humidity and temperature. Pups were organized into adolescent and adult groups based on age at imaging. We regarded the adolescent and adult groups as one group for further analysis as the core symptoms of ASD and comorbidities tend to persist throughout the lifetime^89,90^. We assumed that if there existed distinct characteristics in the brain that represent the autistic-like behaviors, the features would not vary with age as long as the core symptoms persist.

### Three-chamber social approach test

A three-chamber social approach test was performed between postnatal weeks (PNWs) 4 and 6 as described previously with minor modifications^91^. The apparatus is a rectangular, three-chambered box made of transparent Plexiglas with removable floor and partitions. Each chamber was 30 cm L × 60 cm W × 35 cm H. The test consisted of three phases. In the first phase (habituation), a test rat was placed in the center of the empty three-chamber apparatus with two small wire cages in the left or right chamber to habituate for 5 minutes. In the second phase (sociability test), an age- and sex-matched rat (S1) that had never been exposed to the test rat, was placed in one of the two wire cages. The empty wire cage was presented as a novel object (O). Then the two entrances were opened to allow the test rat in the center to freely explore each of the three chambers for 10 minutes. In the third phase (social novelty preference test), the test rat was gently guided to the center chamber, and another age- and sex-matched novel rat (S2) was placed in the empty wire cage. Then the two entrances were opened to allow the test rat in the center to freely explore for another 10 minutes. The apparatus was cleaned thoroughly with 70% ethanol and water between subjects. All tests were performed between 9 AM and 6 PM. Time spent with the nose point of the test rat in the immediate proximity of the wire cage was measured manually.

### ^18^F-FDG PET acquisition and image preprocessing

PET scans were performed at PNW 6 in the adolescent group, and on PNW 62 in adult group. All rats were deprived of food for 12–18 hours before the scanning, but had access to water at all times. The rats were anesthetized with 1.2% isoflurane evaporated in a mixture of 30% O_2_ and 70% N_2_ for 5 minutes, then were injected intraperitoneally with ^18^F-FDG. Injected ^18^F-FDG activity in control males (40.52 ± 0.40 MBq), VPA-treated males (40.12 ± 0.34 MBq), control females (40.48 ± 0.35 MBq), and VPA-treated females (40.48 ± 0.31 MBq) did not vary among groups. All values are mean ± standard error of the mean (SEM). The rats were moved back to the plastic cages, and woken immediately from anesthesia. The cage was placed in a quiet room with dim light for 50 minutes. PET scans were acquired on Siemens Inveon scanner (Siemens, Knoxville, TN, USA). Static emission scan was started at 60 minutes after injection under isoflurane anesthesia. Emission scan was acquired for 20 minutes, followed by 20 minutes of transmission scan. All PET scans were performed between 9 AM and 6 PM. The images were reconstructed using the ordered subsets expectation maximization (OSEM) algorithm with attenuation, scatter, and random correction. The voxel size was 0.776 × 0.776 × 0.796 mm.

Spatial processing was performed using Statistical Parametric Mapping (SPM12, Wellcome Trust Department of Cognitive Neurology, London, UK). All reconstructed images were spatially normalized to the ^18^F-FDG rat brain template (PMOD 3.6, PMOD Technologies Ltd, Zürich, Switzerland). Spatially normalized images were smoothed with a 1.6 × 1.6 × 1.6 mm^3^ full-width-half-maximum (FWHM) Gaussian kernel followed by proportional scaling for global intensity normalization.

### Independent component analysis of cross-sectional PET data

To examine alterations in cross-sectional metabolic networks, we defined region of interests (ROI) in a data-driven way using a cross-sectional ICA modified from our previous study^32,92^ in the following manner. For each PET image (total number *M* = 77, i.e., 17–22 subjects × 2 sexes [male vs. female] × 2 treatment conditions [VPA-treated vs. control]), we extracted a vector of ^18^F-FDG PET intensity from the voxels within the whole brain (total number of voxels, *K* = 370,675). All vectors from the *M* PET images were concatenated to a matrix **x**, which was decomposed into an independent component matrix **s** (N equals the number of independent components) and a mixing matrix **A** using an ICA algorithm. The matrix sizes of **x**, **s** and **A** were (*M* × *K*), (*N* × *K*) and (*M* × *N*).

For each voxel, a vector of *i*-th PET image x_i_ can be composed of *N* independent spatial components (ICs) s_j_, j = 1,.., *N*, as below,

$${x}_{i}=\sum _{j=1}^{N}{a}_{ij}{s}_{j},\,i=1,\ldots ,M$$

where the weight a_ij_ indicates the contribution of source s_j_ to compose **x**_i_. This can be rewritten as,

$${\bf{x}}={[{{\bf{x}}}_{1},\cdots ,{{\bf{x}}}_{{\rm{M}}}]}^{{\rm{T}}}={\bf{A}}{[{{\rm{s}}}_{1},\cdots ,{{\rm{s}}}_{{\rm{N}}}]}^{{\rm{T}}}={\bf{A}}{\bf{s}}$$

where **x**_i_ is a vector of the *i*-th PET image, s_i_ is a *j*-th IC, and **A** is a *M* × *N* mixing matrix composed of weight elements a_ij_. In the current study, the number of ICs (*N*) was chosen after reducing the dimensionality of the data by using a principal component analysis retaining 90% of the explained variance. The mixing matrix **A** can be estimated using ICA algorithms, which maximize the mutual independency between the estimated functional components. To reliably estimate the functional components, we followed an ICASSO framework^93^ using FastICA^94^. In this ICASSO framework, we ran FastICA 100 times with random initial values. From the pool of components driven at each run, ICASSO searches cluster centroids by computing hierarchical clustering according to the dissimilarities among components using average-linkage strategy. These cluster centroids are considered more reliable estimates for independent component^93^.

### Statistical analysis for metabolic weights of independent components

The mixing matrix **A**, driven by ICA algorithms, carries information on the weight (contribution) of each IC to a measured PET image. This weight corresponds to the activity of the component. For the weight element a_ij_ and for the *j*-th IC (*j* = 1,.., *M*) to the *i*-th PET image (*i* = 1,.., *M*), we conducted two-way ANOVA with the factors: treatment condition (VPA-exposed vs. control) and sex condition (male vs. female). Comparisons were performed using permutation tests with 10,000 randomizations, followed by false discovery rate (FDR)^95^ corrections (FDR ≤ 0.05) for multiple tests.

### Metabolic network construction

Each brain region identified by ICA served as nodes throughout the brain. ^18^F-FDG uptake in the region defined by each IC was normalized to global brain activity. In order to construct metabolic networks, we used two types of connectivity definitions; 1) Pearson’s correlation based connectivity and 2) partial correlation-based connectivity. Pearson’s correlation method inherently cannot factor out latent effects of a third and/or fourth node that may modulate connectivity between the two nodes. This makes interpretation of the correlative activities unclear, whether they are from the intrinsic structural connection or from polysynaptic induction, common modulatory effects, or common feed-forward projections via the thalamus^96^. By definition, partial correlation measures connection between two brain regions while controlling for the effects from other brain regions^97^. Thus, partial correlation measures are more efficient in revealing direct associations between brain areas. Therefore, we evaluated both measures in the current study.

For each pair of ICs, Pearson’s correlation coefficient between IC weights was calculated across subjects within each group to generate four correlation matrices, one for each factor, VPA-exposed vs. control, and male vs. female rats. Correlation matrices for each group were transformed to Z scores using a Fisher transformation. Permutation testing was performed to evaluate statistical differences of elements (connectivity) of correlation matrices between groups. The subject label for each group was permuted 10,000 times. A FDR threshold of 0.05 was used to determine significance.

The SICE, a type of graphical lasso (least absolute shrinkage and selection operator), was used to estimate the partial correlation matrix of the total brain network. To determine the optimal sparsity control parameter, we used a stability approach to regularization selection (StARS)^33,34^. We further adopted a constrained optimization algorithm to estimate the strength of connections in the graph obtained by SICE^98^. To test statistical differences caused by VPA exposure and sex, we obtained a null distribution by permuting subject labels and by re-estimating the optimal network for each of the four factors. Then we evaluated interactions between VPA treatment and sex. Main effects were controlled by approximate permutation testing using orthogonal contrasts and subject labels were permuted as previously described^99,100^. The entire subject labels were permuted 10,000 times. A FDR threshold of 0.05 was used to determine significance.

### Sparse canonical correlation analysis

We performed a multivariate analysis using SCCA to explore the relationship between the behavioral data and the IC weights (activities)^101,102^. We selected behavioral parameters such that higher values refer to more normal social interactions. In particular, the greater the length of time spent with the first stranger in the sociability session and the longer period spent preferring the second stranger over the first stranger during the social novelty preference test session reflected more normal social behavior. Thus, we were able to correlate these behavioral parameters with the IC weights using SCCA. No penalty was applied for behavioral parameters, as the number of parameters was two. This procedure produced a canonical variate that described the maximal correlation between behavior and the metabolic activity of ICs.

### Statistical analysis

Statistical analyses were performed using scripts written in house (Matlab, R2016b, Mathworks, Inc. USA). Behavioral data were analyzed with a two-way ANOVA and t-test using permutation testing. The permutation was repeated 10,000 times. We also used a one-way ANOVA with injected 3^18^F-FDG activity as a between-group factor. The null hypothesis was rejected for alpha greater than 5%. Details on statistical method applied in imaging analyses are described in each section.
